# LC-QTOF-MS Characterization, Antioxidant Activity, and *In Vitro* Toxicity of Medicinal Plants from the Tri-Than-Thip Remedy

**DOI:** 10.1155/2022/4477003

**Published:** 2022-01-31

**Authors:** Palika Wetchakul, Piriya Chonsut, Chuchard Punsawad, Sineenart Sanpinit

**Affiliations:** ^1^Department of Applied Thai Traditional Medicine, School of Medicine, Walailak University, Nakhon Si Thammarat 80160, Thailand; ^2^Department of Medical Science, School of Medicine, Walailak University, Nakhon Si Thammarat 80160, Thailand; ^3^Research Center in Tropical Pathobiology, Walailak University, Nakhon Si Thammarat 80160, Thailand

## Abstract

**Background:**

The world population has exhibited increased trust in folk medicine, including Thai folk medicine, for the treatment of various illnesses. However, the comparative antioxidant and cytoprotective activities against oxidative damage of Tri-Than-Thip (Tri-TT), a Thai folk remedy, have not been reported.

**Objectives:**

The purpose of this study was to evaluate the antioxidant capacities of Tri-TT and its herbal constituents, *Cassia fistula*, *Pithecellobium dulce*, and *Ficus benjamina*.

**Methods:**

Extracts were obtained from Tri-TT and its herbal constituents. The free radical scavenging activities, cytotoxicity, ferric-reducing antioxidant power (FRAP), metal chelating activities, total phenolic compound (TPC) contents, and total flavonoid (TF) contents of Tri-TT extract were investigated, and qualitative analysis of the chemical composition of Tri-TT extract was performed by LC-QTOF-MS.

**Results:**

Tri-TT extract exhibited remarkable scavenging activities toward DPPH, ABTS, and superoxide anion radicals, with IC_50_ values of 0.081 ± 0.00, 0.021 ± 0.00, and 0.205 ± 0.057 mg/mL, respectively. The oxygen radical antioxidant capacity (ORAC) and FRAP values of Tri-TT were 6.499 ± 0.67 *μ*M TE/g extract and 1,919.71 ± 63.14 mM FeSO_4_/mg sample, respectively. *P. dulce* had the highest scavenging activities and antioxidant capacity followed by *C. fistula* and *F. benjamina*. The TPC and TF content of Tri-TT extract were 287.87 mg equivalence/g extract and 225.62 mg catechin equivalent/g extract, respectively. The highest TPC was obtained from *P. dulce*, and TF content was observed in *C. fistula*. Using LC-QTOF-MS, a total of 25 compounds were tentatively identified in Tri-TT, including polyphenols such as luteolin, gallic acid, baicalein, apigenin, epicatechin, and ellagic acid. In addition, Tri-TT extract demonstrated nontoxicity (cell viability >90%) to Vero cells at the highest tested concentration of 80 *μ*g/mL.

**Conclusion:**

This study demonstrated that the Tri-TT remedy is a promising candidate as a natural source of antioxidant activity, suggesting that the polyphenol content of plants may contribute to antioxidant activities.

## 1. Introduction

Free radicals include reactive oxygen species (ROS) as well as reactive nitrogen species (RNS). Within cells, ROS function as secondary messengers in intracellular signaling cascades involved in human diseases such as gastric ulcers, hypertension, preeclampsia, neurological disorders, atherosclerosis, inflammatory conditions, certain cancers, and the process of aging [1]. An antioxidant can delay or relieve and inhibit oxidative damage to a target molecule and its ability is to trap free radicals by acting as a free radical scavenger, chelating, and other mechanisms to prevent lipid oxidation, and carbonyl scavengers as a way to avoid lipid oxidation consequences antioxidants. Antioxidants mitigate the effects of free radicals and protect cells from damage. Plants are good sources of antioxidants, and herbs have been used medicinally since ancient times based on reports of folk medicine. Natural sources of antioxidants have been of interest to researchers, as they are inexpensive and natural [2]. In Thailand, many plants used in food and medicine have been reported as sources of natural antioxidants. From the types of remedies described in the Thai Pharmaceutical Textbook, herbal formulations are commonly added to Thai ancient household remedies [3]. Thai remedies have shown antioxidant capacity when studied as an herbal formulation, and subsequent deeper studies on their antioxidant activities revealed good antioxidant activity and a potential for development as natural dietary supplements, including Triphala and Jatu-phala-Tiga (JPT). A previous study reported that JPT has strong antioxidant activities, particularly the water extract of the polyherbal tonic. These findings rationalize further investigation of JPT infusions as a promising agent for antiaging and oxidative stress prevention [4]. The overall report generated interest in Thai drug formulas against free radicals from natural and Tri-Than-Thip (Tri-TT) remedies and is another interesting Thai drug formula.

The Tri-TT remedy is a group of herbs from Thai traditional medicine that have historically been used for nourishing breastfeeding mothers, healing wounds, and relieving diarrhea, and this remedy contains many parts of *Cassia fistula*, *Pithecellobium dulce*, and *Ficus benjamina*, which were reported to have good antioxidant capacity [5–7]. Although a previous study confirmed the good antioxidant ability of different parts of the herbal elements, there have been a few reports on the roots of the component herbs. Therefore, this study aimed to evaluate the antioxidant capacity and cytotoxicity of the Tri-TT remedy and its three individual botanical constituents.

## 2. Materials and Methods

### 2.1. Chemicals

2,2-Diphenyl-1-picrylhydrazyl (DPPH), 2,2′-azobis-2-methyl-propanimidamide, dihydrochloride (AAPH), 2,2,-azinobis [3-ethylbenzothiazoline-6-sulfonic acid]) (ABTS), ethylenediaminetetraacetic acid (EDTA), Trolox, gallic acid, sodium carbonate, hydrogen peroxide, phosphate buffered saline (PBS), nitrotetrazolium blue chloride, iron(II) chloride (FeCl_2_), iron(III) chloride hexahydrate (ferric chloride), magnesium sulfate, and Folin–Ciocalteu reagent were purchased from Sigma-Aldrich (Mumbai, India). Acetic acid and hydrochloric acid were purchased from J. T. Baker (Haryana, India). Aluminum chloride (AlCl_3_), potassium dihydrogen orthophosphate, potassium persulfate (K_2_S_2_O_8_), sodium acetate trihydrate, sodium carbonate anhydrous (Na_2_CO_3_), sodium chloride (NaCl), sodium nitrite (NaNO_2_), sodium hydroxide (NaOH), and sodium dihydrogen orthophosphate were purchased from Ajax Finechem (New South Wales, Australia). Dimethyl sulfoxide (DMSO) was purchased from Fisher Chemical (Chicago, United States). 2,4,6-Tris(2-pyridyl)-s-triazine (TPTZ) and 3-(2-pyridyl)-5,6-diphenyl-1,2,4-triazine-4′,4′-disulfonic acid sodium salt (ferrozine) were purchased from Fluka (Buchs, Switzerland). Solvents, including ethanol and methanol, were obtained from Merck (Darmstadt, Germany). All reagents and chemicals used were of analytical reagent grade and purchased from commercial sources. Deionized water was used for sample preparation, dilution, and rinsing apparatus prior to analysis.

### 2.2. Preparation of the Tri-TT Remedy and Three Botanical Extracts

One kilogram of dried root powders of *P. dulce*, *C. fistula*, and *F. benjamina* was mixed together in a 1 : 1:1 ratio to obtain Tri-TT. Briefly, both Tri-TT and individual plant ingredient powders were individually macerated with 1000 mL of ethanol at room temperature for 3 days, filtered through Whatman No. 1 filter paper, and dried using a vacuum rotary evaporator (Heidolph, Germany). All extracts were stored at 20°C until further experiments. The extraction yield of each plant extract was calculated as weight percent (% w/w) [4]:

(1) $$\begin{array}{c} \text{Extraction yield }\left(\%\right)=\frac{\text{weight of the dry extract}}{\text{weight of the initial dry material}}\times 100. \end{array}$$

### 2.3. Free Radical Scavenging Activities

The antioxidant activity of the extracts was evaluated using the DPPH and ABTS assays described by Ghasemi Pirbalouti et al. [8]. For DPPH radical scavenging assays, 1 mL of sample extract was diluted in 2-fold increment to various concentrations (1.22 to 2500 *μ*g/mL), and 20 *μ*L of each sample at different concentrations was placed in a 96-well plate containing 80 *μ*M DPPH in ethanol solution (180 *μ*L). The 96-well plate was incubated in the dark for approximately 30 min at room temperature. The absorbance of the solution was read at 520 nm. Trolox was used as a positive control and used to construct a calibration curve, and half maximal inhibitory concentration (IC_50_) values were calculated.

To generate ABTS^+^, 2 mM ABTS and 2.45 mM potassium persulfate were mixed together at a volume ratio of 1 : 1, and then the mixture was stored in the dark at room temperature for 16 h. The absorbance of the solution was maintained at 0.70 ± 0.05 at 734 nm. Sample extracts (10 *μ*L) at various concentrations (between 1.22 and 2500 *μ*g/mL) were added to a 96-well plate, followed by the addition of 1 mL of ABTS^+^ solution and incubation for 6 min. The absorbance was read at 734 nm. Trolox was used as a positive control and used to construct a calibration curve. Finally, the scavenging activity is expressed as the concentration that caused 50% inhibition of ABTS^+^, as in the DPPH assay:

(2) $$\begin{array}{c} \text{scavenging activity }\left(\%\right)=\frac{\left[Ab_{\text{control}}-\;Abs_{\text{sample}}\right]\times 100}{Abs_{\text{control}}}. \end{array}$$

### 2.4. Metal Chelating Activity

The ability of the polyherbal extracts to chelate ferrous ions was measured by a previously described colorimetric metal chelating activity (MCA) method [9]. Briefly, 0.1 mM FeSO_4_ (0.2 mL) and 0.25 mM ferrozine (0.4 mL) were added to 0.2 mL of plant extract with a concentration range of 0.03 to 62.50 mg/mL. After incubation at room temperature for 10 min, an increase in the absorbance of the stable ferrous-ferrozine complex was detected at 562 nm. EDTA was used as a positive control. MCA was calculated using

(3) $$\begin{array}{c} \text{metal chelating activity }\left(\%\right)=\frac{\left[Ab_{\text{control}}-\;Abs_{\text{sample}}\right]\times 100}{Abs_{\text{control}}}. \end{array}$$

### 2.5. Single Electron Transfer-Based FRAP Assay

The ferric-reducing antioxidant power (FRAP) activity of the plant extract was determined according to a previous study with minor modifications [10]. The FRAP working solution was freshly prepared by mixing 10 mL of 300 mM acetate buffer, 1 mL of 10 mM TPTZ solution, and 10 mL of 20 mM ferric chloride. Twenty milliliters of each extract was diluted in ethanol to various concentrations of 0.625 to 1.35 mg/mL, added to each well in a 96-well microtiter plate, and incubated at room temperature for 30 min in the dark. The absorbance of the solution was detected at 562 nm by the colored product from an intense blue color complex formed by the reduction of TPTZ to ferrous-TPTZ in the presence of electron donating antioxidants at low pH, and the reducing capacity is expressed as *μ*M Fe_2_SO_4_/mg extract.

### 2.6. Superoxide Anion Radical Scavenging Activity

Superoxide anions can be found in the process of energy production in cells in the body, which leads to lipid peroxidation. Therefore, the superoxide anion scavenging ability and the capacity to reduce the rate of lipid peroxidation were evaluated. This activity was evaluated based on the reduction of NBT according to a previous report with minor modifications [11]. The riboflavin/methionine/illuminate system was used to generate superoxide anion radicals, which reduced NBT to form purple formazan (NBT^2+^).

The reaction mixture contained 100 *μ*L of NBT (400 *μ*g/mL) and 0.4 mL of a solution consisting of riboflavin (30 *μ*g/mL), methionine (30 *μ*g/mL), EDTA (20 *μ*g/mL), and the plant extract at different concentrations (2-fold dilution; 4.88 to 156.25 *μ*g/mL) diluted in 0.05 M PBS (pH 7.4). Photoinduced superoxide radicals were initiated with illumination by a fluorescent lamp (20 W) at 25°C for 25 min. After incubation, the absorbance was measured at 560 nm. The scavenging activity is expressed as the concentration that caused 50% inhibition of superoxide anion radicals (IC_50_; mg/mL). Catechin was used as a reference compound.

### 2.7. Hydrogen Atom Transfer-Based Assay and Peroxyl Radical Scavenging Assay (ORAC Assay)

An oxygen radical antioxidant capacity (ORAC) assay with some modifications was used to evaluate the antioxidant activity of the extracts against peroxyl radicals generated from the thermal homolysis of AAPH [12]. The assay was carried out in black-walled 96-well plates with PBS (pH 7.4). The standard was 25 mL of Trolox solution, and the samples were analyzed as 25 mL solutions at various concentrations of 0.2 to 100 *μ*g/mL (2-fold dilution). All experimental wells received 150 mL of sodium fluorescein (40 nM). After 30 min of incubation at 37°C, 25 *μ*L of AAPH solution was added to the solution. The plate was placed into a microplate reader and analyzed with an excitation wavelength of 485 nm and emission wavelength of 535 nm, every 5 min for 90 min. The antioxidant capacity is expressed as Trolox equivalents per *μ*g of extract (*μ*M TE/*μ*g E). This activity was calculated using equations (4) and (5).

(4) $$\begin{array}{c} \text{AUC}=\left(\frac{R_{1}}{R_{1}}\right)+\left(\frac{R_{2}}{R_{1}}\right)+\left(\frac{R_{3}}{R_{1}}\right)+\cdots +\left(\frac{R_{n}}{R_{1}}\right), \end{array}$$

where *R*_1_ is the fluorescence reading at the initiation of the reaction and *R*_*n*_ is the last measurement.

(5) $$\begin{array}{c} \text{Net AUC}={\text{AUC}}_{\text{sample}}-{\text{AUC}}_{\text{blank}}. \end{array}$$

### 2.8. Determination of TPC Content

The total phenolic compound (TPC) content in plant extracts was determined according to a previous study with minor modifications [13]. Briefly, 120 *μ*L of extract (2.5 mg/mL) was mixed with 1 mL of Folin-Ciocalteu reagent for 5 min. Then, 1 mL of 20% w/v sodium carbonate solution was homogenously mixed and allowed to stand for 90 min in the dark at ambient temperature. Then, the absorbance was measured at 725 nm. The TPC content was nitrotetrazolium determined with a calibrated curve of gallic acid and is expressed in terms of milligrams of gallic acid equivalents per gram of extract.

### 2.9. Determination of TF Content

To determine the total flavonoid (TF) content, the plant extracts were analyzed based on a previous study with minor modifications [14]. Briefly, 50 *μ*L of the plant extract (2.5 mg/mL) was combined with 300 *μ*L of 5% (w/v) sodium nitrite, 300 *μ*L of 10% (w/v) aluminum trichloride, and 4 mL of distilled water, after which the solution was homogenously mixed and incubated for 6 min at ambient temperature. The reaction was stopped with 2 mL of 1 M sodium hydroxide after 5 min. The absorbance was recorded at 510 nm. The TF content is expressed as catechin equivalents per gram dry matter.

### 2.10. MTT Assay

The plant extracts were tested for *in vitro* cytotoxicity using Vero cells by a 3-(4,5-dimethylthiazol-2-yl)-2,5-diphenyltetrazolium bromide (MTT) assay [15]. Cells (1 × 10^5^/well) were placed in 96-well plates and incubated at 37°C with 5% CO_2_ for 24 h. Then, different concentrations of samples at 5 to 80 *μ*g/mL (2-fold dilution) were added and incubated for 24 h. Each sample was analyzed in triplicate. After sample incubation, 100 *μ*L/well 5 mg/mL 0.5% MTT was added to the wells and incubated for 4 h. When purple precipitate was clearly visible under a microscope, 100 *μ*L of DMSO was added, and the plate was shaken for 5 min. The absorbance of each well was measured at 540 nm with a microtiter plate reader using DMSO as a blank, and percentages of cell viability were calculated:

(6) $$\begin{array}{c} \text{percentage of cell viability}=\frac{\text{absorbance of extract treated wells}}{\text{absorbance of untreated wells}}\times 100. \end{array}$$

### 2.11. Liquid Chromatography-Quadrupole Time-of-Flight Mass Spectrometry (LC-QTOF MS) Conditions

The composition of Tri-TT extract was analyzed by UHPLC with a column from Zorbax Eclipse Plus C18 Rapid Resolution HD column (150 mm length^*∗*^ 2.1 mm inner diameter, particle size 1.8 *μ*m), using a liquid chromatograph-quadrupole time-of-flight mass spectrometry (LC-QTOF MS) instrument (1290 Infinity II LC-6545 Quadrupole-TOF, Agilent Technologies, USA). The temperature was maintained at 40°C, and the injection volume was 2 *μ*L. Elution was performed with the following 30 min, and mobile phase program was as follows: A: 0.1% formic acid in water, B: acetonitrile, and flow rate: 0.2 mL/min. LC–MS/MS analysis was performed in negative ion mode with a scanning range from m/z 100 to 1500 using a Dual AJS ESI ion source.

### 2.12. Statistical Analysis

The data are presented as the mean value ± SD value. One-way ANOVA was conducted. Minitab software was used to calculate the significant differences (*p* < 0.05) between mean values.

## 3. Results and Discussion

### 3.1. Extract Yield

Our results demonstrated that the yield of the ethanol extract of Tri-TT was 1.489%. The highest extraction yield was found for *C. fistula* (2.670%), followed by *P. dulce* and *F. benjamina* (Table 1). This finding was consistent with that of previous studies, showing that alcohol extracts have a good percentage yield [16–18].

### 3.2. Free Radical Scavenging Activities, DPPH Assay, and ABTS Assay

DPPH and ABTS assays are the most commonly used antioxidant assays and are spectrophotometric techniques based on quenching of stable-colored radicals [18]. The DPPH radical scavenging activity and ABTS radical content of the Tri-TT extract were 0.081 ± 0.00 mg/mL and 0.021 ± 0.00 mg/mL, respectively. For the herbal components, *P. dulce* had remarkable DPPH and ABTS free radical scavenging activities, with IC_50_ values of 0.07 ± 0.00 and 0.10 ± 0.00 mg/mL, respectively (Table 2). This study is the first to examine the antioxidant capacity of the Tri-TT remedy. A previous study on herbal components showed that the methanol extract of the bark and leaves of *P. dulce* demonstrated antioxidant capacities by the DPPH assay of 150.23 ± 2.8 and 250.32 ± 4.8 *μ*g/mL, respectively [16]. The radical scavenging activity determined using the DPPH radical assay yielded IC_50_ values of 5.20 ± 0.15, 88.29 ± 2.65, 87.39 ± 2.60, and 96.49 ± 2.90 *μ*g/mL for petroleum ether, ethyl acetate, aqueous, and methanol extracts of *P. dulce* fruit, respectively [13]. The percentages of inhibition for methanol extracts of *C. fistula* bark at concentrations of 20, 40, 60, 80, and 100 *μ*g/mL were 8.88, 27.84, 40.11, 58.88, and 85.82, respectively [19]. In addition, the ethanolic *F. benjamina* leaf extract had an inhibition percentage of 44.87 and was usually nontoxic [20]. The DPPH and ABTS assays showed that the *P. dulce* extract had the strongest antioxidant activity, followed by *C. fistula* and *F. benjamina* (Table 2). The results of this study were similar to those of Selvakumar, who reported that the ethanol extract of flowers from these species had an IC_50_ value of 85.20 *μ*g/mL [21]. The 50% ethanol extract of *C. fistula* flowers exhibited an antioxidant activity of 47% at 4 *μ*g/mL, and the methanol extract of *F. benjamina* leaves demonstrated higher antioxidant potential with a significant IC_50_ value of 37.76 at 100 *μ*g/mL [22, 23]. Based on the antioxidant activity results from the above report, Tri-TT and its herbal components show good potential to control oxidative stress by the DPPH and ABTS assays, but both methods simulate free radical formation at the *in vitro* level and generate free radicals not found in the body. Therefore, the NBT assay and ORAC assay are important techniques that will confirm the antioxidant capacity of an extract. The superoxide anion radical scavenging activity was estimated by the NBT method [24]. Superoxide radicals are known to be very harmful to cellular components as precursors of more ROS, and the ability of a plant extract to scavenge oxidation and mitigate biological damage is of interest [25]. The superoxide radical scavenging activity was defined as the concentration that produced 50% inhibition of superoxide anion radicals, as shown in Table 2. The IC_50_ value of Tri-TT extract was 20.05 ± 0.057 mg/mL, similar to that of the Triphala remedy, which is a popular tonic drug, and antioxidants from a traditional Ayurvedic herb remedy showed an IC_50_ value of 42.95 ± 2.07 *μ*g/mL [26]. The herbal component *C. fistula* showed the best antioxidant activity of 0.08 ± 0.02 mg/mL. As previously reported, the IC_50_ values of ethanolic and water *C. fistula* fruit extracts were compared, and the *C. fistula* ethanol extract showed the best NO radical scavenging activity of 1,232.64 ± 1.73 *μ*g mL [6].

**Figure 1:**
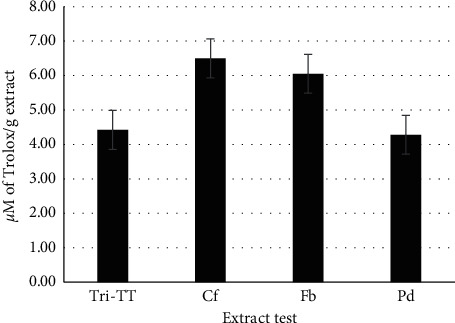
Effects of Tri-Than-Thip (Tri-TT), *Cassia fistula* (Cf), *Ficus benjamina* (Fb), and *Pithecellobium dulce* (Pd) ethanol extracts on the production of peroxyl radicals. All values are presented as the means ± SD. Bars with different letters indicate statistically significant differences among groups at *p* < 0.05 by one-way ANOVA.

According to the ORAC assay, which has been widely used to investigate the scavenging activities of several natural compounds, hydroxyl radicals are major active oxygen species causing lipid peroxidation and enormous biological damage [20, 27]. The tested extracts scavenged peroxyl radicals in a concentration-dependent manner, as indicated by the inhibition of fluorescence decay. In this study, *C. fistula* had remarkable peroxyl radical scavenging properties with an ORAC value of 6.499 ± 0.67 *μ*M TE/g extract (Figure 1). A previous report demonstrated that the IC_50_ value of an ethanolic *C. fistula* extract from flowers showed a better hydroxyl radical scavenging activity (IC_50_) of 609.03 ± 0.64 *μ*g/mL than that of the aqueous *C. fistula* fruit extract, which exhibited a moderate activity of 1748.86 ± 0.65 *μ*g/mL. The above activities of the *C. fistula* ethanol and water extracts clearly indicate strong concentration-dependent activity [6], and it may be concluded that *C. fistula* was the main component of Tri-TT involved in preventing and reducing intracellular ROS levels.

### 3.3. In Vitro Metal Chelating and FRAP Radical Scavenging Activity

The FRAP assay is relatively simple and easy to conduct. The FRAP assay measures the potential of antioxidants to reduce the ferric tripyridyl triazine (Fe3+-TPTZ) complex and produce a blue ferrous complex [27]. The ability of a compound to reduce iron(III) to iron(II) generally depends on the presence of reductants [28], which exhibit antioxidative potential by quenching the free radical chain and donating a hydrogen atom [29, 30]. The results (Table 2) indicated that Tri-TT had the highest MCA with an IC_50_ value of 0.02 ± 0.00 mg/mL. The ion-chelating effect increased with increasing concentrations and stimulated a remarkable reducing power, with an FRAP value of 1,919.71 ± 63.14 mM FeSO_4_/mg (Table 3). The value in this study was higher than that reported in a previous study on traditional Thai remedies. Ya-hom Intajak and Jatu-Phala-Tiga remedies had FRAP values of 0.93 ± 0.12 (mmol FeSO_4_/g) and 23.07 ± 1.84 (mM FeSO_4_/mg), respectively [4, 31]. In addition, *P. dulce* possessed the highest reducing power, with an FRAP value of 3,335.38 ± 439.75 mMFeSO_4_/mg, whereas its MCA IC_50_ value was 0.01 ± 0.00 mg/mL. Previous reports showed that the FRAP IC_50_ value of a *P. dulce* methanol extract was 13.70 *μ*g/mL [7] and that the extract possessed antioxidant, antibacterial, and antifungal activities [32]. Thus, it was found that Tri-TT extract was a good choice for antioxidant use according to the FRAP and MCA assay results.

### 3.4. TPC and TF Contents

TPC and TF contents are indicators widely used to represent antioxidant activity. The high potential of phenolic and flavonoid compounds to scavenge radicals may be explained by their phenolic hydroxyl groups [33]. This study determined the TPC and TF contents of the Tri-TT remedy and individual botanical extracts. The TPC contents and TF contents were 287.87 ± 15.10 mg equivalence/g extract and 225.62 ± 2.056 mg catechin equivalent/g extract, respectively. In recent years, researchers have been interested in the search for new, natural antioxidants. Tri-TT has good antioxidant properties, similar to Tri-phal, Tri-chin-Tha-La-Ma-Ka, Tri-Ke-Son-Mat, Tri-Sa-Mo, Tri-Ti-Pa-Ya-Ros, and Tri-Su-Ra-Pon, and previous studies have described Tri-TT as the best antioxidant source among Tri-remedy groups from Thai folk medicine [3, 19]. In addition. Tri-TT has higher TPC and TF contents than Ya-hom Intajak and twenty polyherbal remedies either with rejuvenating effects or that are used as health-promoting tonics [31, 34]. This study detected significant differences (*p* > 0.05) in the TPC contents of the three botanical extracts and showed that *P. dulce* had the highest TPC content, similar to the methanol extract of leaves and bark, which exhibited a TPC content of 0.084 ± 0.24 0.129 ± 0.11 *μ*g/mL gallic acid equivalents [16]. The *C. fistula* extract exhibited the highest TF content, consistent with a previous study that reported TF contents of methanolic *C. fistula* leaf and stem extracts of 45.08 ± 1.37 and 4.17 ± 0.20 (quercetin equivalent) mg/g extracted compound, respectively [35]. In 2002, researchers reported the TPC and TF contents of several parts of *C. fistula*, including young leaves, old leaves, twigs, bark, flower buds, flowers, and pods [36], but this study showed that the roots have higher TPC and TF contents (Table 3). From all the experimental reports above, the results differed due to differences in the reactions and mechanisms of the methods used, which should be considered when comparing the antioxidant activities of plant extracts and compounds in herbs [37].

### 3.5. Cytotoxicity Analysis by the MTT Assay

**Figure 2:**
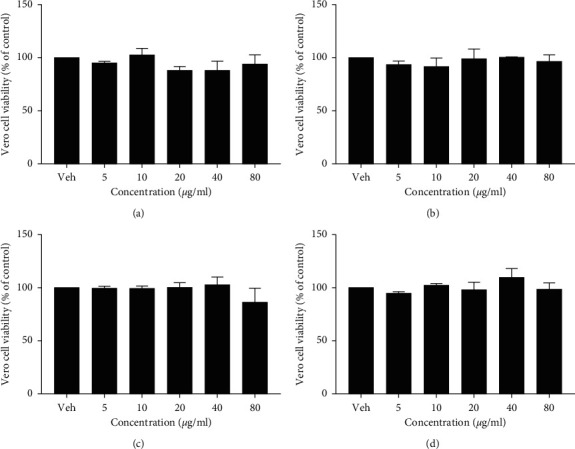
Cytotoxicity of Tri-TT (a), *Ficus benjamina* (b), *Cassia fistula* (c), and *Pithecellobium dulce* (d) extracts on Vero cells. The value was from three replicates (*n* = 3).

Vero cells, also known as African green monkey kidney cells, are recognized by the World Health Organization (WHO) and Chinese Pharmacopoeia in producing vaccines [38]. In the present study, the cytotoxic effects of extracts of Tri-TT and its three botanical extracts were determined by the MTT assay.  Figure 2 shows the viability of Vero cells after treatment with various concentrations of the extracts of Tri-TT and its three botanical extracts. The IC_50_ value indicated the concentration that can inhibit 50% cell proliferation and showed that the extracts had cytotoxic ability. A relatively high IC_50_ means that the compound is more nontoxic to the cell. Similar to a previous report about three-component herbal remedies, Vero cells were exposed to quercetin 3-O-rutinoside, kaempferol 3-O-rutinoside, and kaempferol 3-O-robinobioside from the ethanol extract of *F. Benjamina* leaves, and antiviral activity was evaluated by the plaque assay and exhibited low toxicity [39]. In addition to normal cell testing, herbal components have also been found to inhibit cancer cells and have shown varying activities from toxic to safe. *P. dulce* bark and leaf lipophilic fractions were assessed for their cytotoxic activity using an MTT cell viability assay against two different cancer cell lines, namely, hepatocellular carcinoma and colon carcinoma cells, and the lipophilic extract was reported to possess significant cytotoxic activity in a colon carcinoma cancer line [40], and it was shown that there was cytotoxic potential of *P. dulce* leaf extracts on breast cancer cells (MCF-7 cell line) at 400 mg/mL [41]. In addition, a *C. fistula* methanol extract reduced prostate human cancer cell line viability in a dose-dependent manner in the MTT assay. The lowest viability of cancer cells was observed with 30 *μ*g, at 5.06%, and the vehicle control showed 97.77% cell viability [42].

### 3.6. Liquid Chromatography-Quadrupole Time-of-Flight Mass Spectrometry (LC-QTOF-MS) Conditions

**Figure 3:**
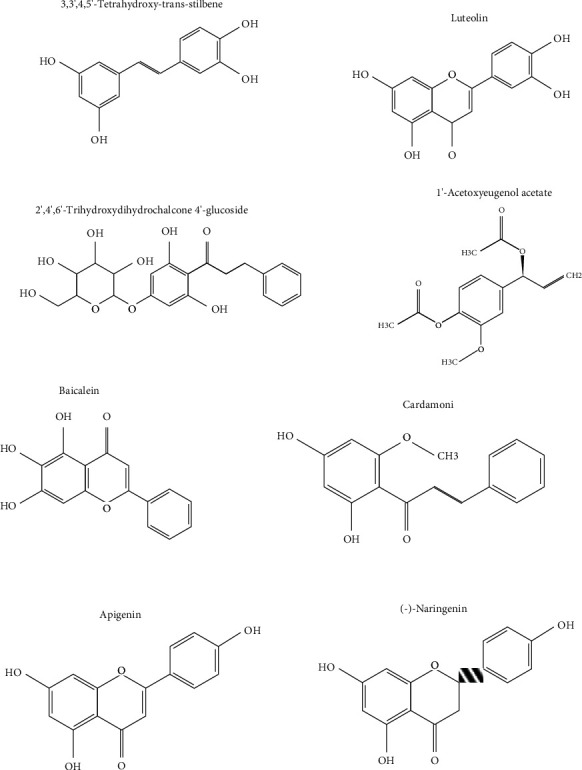
Chemical structure of eight compounds tentatively identified by LC-QTOF-MS.

The qualitative analysis of compounds in the Tri-TT remedy infusion using LC-QTOF-MS in negative mode revealed chemical constituents and the known antioxidant-related constituents, with phenolic acids and flavonoids being the major components. Within the phenolic acid group, catechins such as epicatechin (RT = 6.76) and epigallocatechin (RT = 6.19) were identified, and flavonoids, one of the most widespread groups of plant phenolics, were the main class of compounds characterized in samples and included baicalein (RT = 19.78), genkwanin (RT = 17.43), (-)-naringenin (RT = 13.74), pinocembrin (RT = 17.43), hesperetin (RT = 14.09), luteolin (RT = 12.36), apigenin (RT = 13.71), quercetin 3′-O-glucuronide (RT = 7.28), quinic acid (RT = 2.05), and gallic acid (RT = 3.93). The other detected compounds were 3,3′,4,5′-tetrahydroxy-trans-stilbene (RT = 10.14), 3,3′,4′,5,7-pentahydroxyflavan(4->8)-3,4′,5,7-tetrahydroxyflavan (RT = 8.52), and other chemicals in Table 4. The LC-QTOF-MS analysis showed a high content of flavonoids, which have a very strong antioxidant ability. Flavonoids are the main class of phenolic compounds responsible for antioxidant and free radical scavenging properties. Previous studies on the chemical GC-MS analysis of methanol extracts of *F. benjamina* from leaves and bark identified 28 alkaloids in leaves and 14 alkaloids in bark. A positive correlation with total alkaloid content was observed, suggesting that the level of antioxidant activity in this species is strongly correlated to the alkaloid content [43]. Other parts of *F. benjamina* (stems and roots) contained antioxidant-related chemical constituents, such as methenamine (RT = 2.297), hexadecanoic acid (RT = 10.26), methyl-2-phenylindole (RT = 10.63), 9,12-octadecadienoic acid (RT = 11.91), and palmitic acid (RT = 11.04) [5]. The chemical screening of the marker components in the fruit of *C. fistula* by HPLC showed the chemical structure of 14 compounds, for example, catechin (RT = 13.057), epicatechin (RT = 16.667), quercitrin (RT = 42.038), rutin (RT = 29.601), sennoside B (RT = 36.768), sennoside A (RT = 42.479), and rhein (RT = 89.860) [44]. The last herb component, *P. dulce*, contained many chemical groups with antioxidant effects that were similar to those of the two previous herbs, such as glycosylated compounds and flavonoids (quercetin-3-glucoside, luteolin-7-O-glucoside, and kaempferol-3-O-rhamnoside) as well as fatty acids (azelaic acid, etc.) [45]. The chemicals found in the herbal components and Tri-TT remedy belonged to similar chemical groups as the primary and secondary metabolite constituents of plant parts, with an emphasis on phenolic compounds and flavonoids with potent antioxidant activities (Figure 3). This paper also assessed the antioxidant and free radical abilities of plant parts.

## 4. Conclusions

This study showed that the Tri-TT remedy and its three botanical constituents have complicated chemical constituents according to LC-QTOF-MS fingerprint analysis, as well as remarkable antioxidant, superoxide radical scavenging, hydroxyl radical scavenging, cytotoxic and protective activities against induced oxidative stress in the body. The ethanol extract of Tri-TT could be a significant material for the prevention of several diseases and could be considered a good antioxidant source in the pharmaceutical industry.

## Figures and Tables

**Table 1:** Ethanol extraction yields of the Tri-Than-Thip remedy and the herbal components.

Plant materials	Extraction yield (g/100 g of dried plant materials)
Tri-Than-Thip	1.489
*P. dulce*	2.223
*C. fistula*	2.670
*F. benjamina*	2.051

**Table 2:** Metal chelating activity (MCA) and free radical scavenging capacities of different extracts of Tri-Than-Thip and three botanical extracts.

Extracts	MCA assay (IC_50_; mg/mL)^*∗*^	Radical scavenging properties (IC_50_; mg/mL)^*∗∗*^		
		DPPH	ABTS	NBT
Tri-TT	0.02 ± 0.00	0.08 ± 0.00	0.02 ± 0.00	0.205 ± 0.057
*P. dulce*	0.01 ± 0.00^b^	0.07 ± 0.00^a^	0.01 ± 0.00^a^	0.33 ± 0.23^b^
*C. fistula*	0.02 ± 0.00^c^	0.10 ± 0.00^a^	0.01 ± 0.00^a^	0.08 ± 0.02^a^
*F. benjamina*	0.01 ± 0.00^a^	1.69 ± 1.79^b^	0.06 ± 0.01^b^	1.06 ± 0.25^c^

^*∗*^IC_50_ of EDTA (a positive control) was 0.01 ± 0.00 mg/mL. ^*∗∗*^The IC_50_ values of Trolox obtained from the DPPH, ABTS, and NBT assays were 0.025, 0.020, and 0.025 mg/mL, respectively. ^a–c^Values in the same column with different superscripts are significantly different (*p* < 0.05). Extraction yield (g/100 g dried plant material).

**Table 3:** Ferric-reducing antioxidant power (FRAP), total phenolic compound content, and total flavonoid content of different extracts of Tri-Than-Thip and its three botanical constituents.

Extracts	FRAP assay (mM FeSO_4_/mg)	Total contents (mg equivalent/g of extract)	
		Phenolic compounds	Flavonoids
Tri-TT	1919.71 ± 63.14	287.87 ± 15.10	225.62 ± 2.056
*P. dulce*	3335.38 ± 439.75^a^	368.43 ± 4.71^a^	152.02 ± 46.80^b^
*C. fistula*	1132.89 ± 129.17^b^	278.87 ± 7.03^b^	238.33 ± 16.65^a^
*F. benjamina*	350.26 ± 10.77^c^	66.96 ± 4.77^c^	72.89 ± 6.17^c^

^a–c^Values in the same column with different superscripts are significantly different (*p* < 0.05).

**Table 4:** Compounds identified in the Tri-TT remedy by LC-QTOF-MS.

No.	M/Z	RT (min)	Compounds	Molecular formula	Molecular weight
1	243.07	10.14	3,3′,4,5′-Tetrahydroxy-trans-stilbene	C_14_H_12_O_4_	244.07
2	561.14	8.52	3,3′,4′,5,7-Pentahydroxyflavan(4->8)-3,4′,5,7-tetrahydroxyflavan	C_30_H_26_O_11_	562.15
3	419.14	13.82	2′,4′,6′-Trihydroxydihydrochalcone 4′-glucoside	C_21_H_24_O_9_	420.14
4	285.04	12.36	Luteolin	C_15_H_10_O_6_	286.05
5	263.09	9.79	1′-Acetoxyeugenol acetate	C_14_H_16_O_5_	264.10
6	269.05	19.78	Baicalein	C_15_ H_10_ O_5_	270.05
7	269.08	18.22	Cardamonin	C_16_H_14_O_4_	270.09
8	109.03	5.62	Hydroquinone	C_6_H_6_O_2_	110.04
9	269.05	13.71	Apigenin	C_15_H_10_O_5_	270.05
10	271.06	13.74	(-)-Naringenin	C_15_H_12_O_5_	272.07
11	477.07	7.82	Quercetin 3′-O-glucuronide	C_21_H_18_O_13_	478.08
12	191.06	2.05	Quinic acid	C_7_H_12_O_6_	192.06
13	289.07	6.76	Epicatechin	C_15_H_14_O_6_	290.08
14	285.04	14.01	Kaempferol	C_15_H_10_O_6_	286.05
15	283.06	17.43	Genkwanin	C_16_H_12_O_5_	284.07
16	255.07	17.43	Pinocembrin	C_15_H_12_O_4_	256.07
17	169.01	3.93	Gallic acid	C_7_H_6_O_5_	170.02
18	137.02	6.98	3,4-Dihydroxybenzaldehyde	C_7_H_6_O_3_	138.03
19	305.07	6.19	Epigallocatechin	C_15_H_14_O_7_	306.07
20	471.35	20.57	Corosolic acid	C_30_H_48_O_4_	472.35
21	301.00	9.05	Ellagic acid	C_14_H_6_O_8_	302.01
22	463.09	8.97	Quercetin 3-galactoside	C_21_H_20_O_12_	464.10
23	471.35	23.28	Maslinic acid	C_30_H_48_O_4_	472.35
24	305.18	17.56	Capsiate	C_18_H_26_O_4_	306.18
25	301.07	14.09	Hesperetin	C_16_H_14_O_6_	302.08

## Data Availability

The data that support the findings of this study are available on request from the corresponding author.
